# Potential Involvement of PI3K/AKT Signaling Pathway in the Protective Effects of *Rhinacanthus nasutus* Against Diabetic Nephropathy-Induced Oxidative Stress

**DOI:** 10.3390/antiox15020252

**Published:** 2026-02-14

**Authors:** Junyu Liu, Yehao Lin, Xudong Yi, Min Zhang, Pharkphoom Panichayupakaranant, Joseph Buhagiar, Haixia Chen

**Affiliations:** 1 Tianjin Key Laboratory for Modern Drug Delivery and High-Efficiency, School of Pharmaceutical Science and Technology, Faculty of Medicine, Tianjin University, Tianjin 300072, China; junyuliu@tju.edu.cn (J.L.); linyehao@tju.edu.cn (Y.L.); yxd@tju.edu.cn (X.Y.); 2 China-Russia Agricultural Processing Joint Laboratory, Tianjin Agricultural University, Tianjin 300384, China; 3 State Key Laboratory of Nutrition and Safety, Tianjin University of Science & Technology, Tianjin 300457, China; 4 Phytomedicine and Pharmaceutical Biotechnology Excellence Center, Faculty of Pharmaceutical Sciences, Prince of Songkla University, Hat-Yai 90112, Songkhla, Thailand; pharkphoom.p@psu.ac.th; 5 Faculty of Science, Biomedical Sciences Building, University of Malta, MSD 2080 Msida, Malta; joseph.buhagiar@um.edu.mt

**Keywords:** *Rhinacanthus nasutus*, diabetic nephropathy, natural antioxidants, oxidative stress, PI3K/AKT signaling pathway, glucose homeostasis

## Abstract

Oxidative stress is a primary driver of diabetic nephropathy (DN), highlighting the urgent need for potent natural antioxidants. This study explored the reno-protective potential and associated mechanisms of *Rhinacanthus nasutus* aqueous extract (AE). Phytochemical profiling via Q Exactive HF Orbitrap LC–MS/MS and serum pharmacochemistry analysis identified 38 constituents, among which 25 bioavailable constituents (e.g., caffeic acid and naringenin) might be the key bioactive ones. In the L6 myotubes in vitro assays, AE (75 μg/mL) was observed to upregulate the PI3K/AKT and GLUT4 signaling cytokines, coinciding with enhanced glucose uptake, as confirmed by Western blot with insulin as a positive control. Furthermore, in STZ-induced DN rats, AE could reduce MDA levels (0.58 vs. 1.44 nmol/mgprot) and restore T-SOD, CAT, and GSH-Px levels (170.57, 51.93, 63.68 vs. 114.93, 40.84, 50.99 mgprot). The protective effects were accompanied by the modulation of PI3K/AKT/mTOR signaling axis. These findings suggest that AE exerts dual efficacy involving glucose uptake regulation and oxidative stress inhibition. Consequently, *Rhinacanthus nasutus* represents a promising natural antioxidant resource with potential for the management of DN.

## 1. Introduction

Diabetic nephropathy (DN) is a serious microvascular complication of diabetes. Its pathological features include glomerular ultrafiltration, podocyte damage, and progressive proteinuria, which may eventually lead to end-stage renal disease [1,2]. Recent studies have shown that the progression of DN is driven by a vicious cycle between glucose metabolism disorders and oxidative stress: high glucose load within cells (glycotoxicity) induces excessive production of reactive oxygen species (ROS), and continuous oxidative stress further exacerbates renal structural damage [3,4]. Current therapeutic paradigms for DN have evolved from intensive glycemic control toward multi-target organ protection. Beyond traditional RAAS inhibition, the clinical emergence of SGLT2 inhibitors and GLP-1 receptor agonists has established a dual-benefit framework for metabolic regulation and direct renoprotection [5]. Contemporary research now focuses on precision interventions targeting oxidative stress, inflammatory cascades, and podocyte restoration to arrest fibrotic progression [6,7]. This shift toward holistic yet mechanistic management provides a robust theoretical foundation for exploring natural bioactive compounds as potent adjuncts in DN therapy. Therefore, finding natural bioactive resources that can simultaneously regulate glucose levels and have strong antioxidant properties has become a key intervention strategy for delaying the progression of DN.

*Rhinacanthus nasutus* (L.) Kurz (*R. nasutus*) is a traditional herb in Southeast Asia and southern China. It originated from the National Herbal Medicine Assembly and has the functions of clearing heat, promoting diuresis and soothing the liver. According to the Flora of China, the branches and leaves of the *R. nasutus* can treat cough and hypertension. Additionally, it was used for senile hypertension, diabetes, and arteriosclerosis [8]. In modern pharmacology, it is used to treat diabetes, hypertension, and related inflammatory diseases [8,9,10,11]. However, while its traditional efficacy is well-recognized, rigorous clinical evaluations are imperative to substantiate its pharmacological applications and ensure safety in the context of diabetic complications. Previous studies have indicated that its organic solvent extract and compounds have certain biological activity. Nevertheless, the aqueous extract of *R. nasutus* (AE) remains the most prevalent form of administration in folk medicine. However, the pharmacological substance basis and mechanism of action of AE are unknown till now. In particular, whether AE can synergistically regulate glucose uptake and oxidative stress balance through specific signaling pathways needs to be investigated, which will help in its clinical translation in the context of evidence-based medicine.

This study aimed to systematically evaluate the protective effects and molecular mechanism of AE on DN by adopting an integrated pharmacological approach. Firstly, the active components of AE that enter the bloodstream were screened by using Q Exactive HF Orbitrap LC–MS/MS analysis combined with serum pharmacokinetic technology; subsequently, through network pharmacology, the core action pathways were predicted; finally, the amelioration effects of AE on oxidative stress, regulation of the PI3K/AKT signaling pathways were clarified by using in vitro glucose uptake models and in vivo STZ-induced DN rat models. This study will provide a scientific basis for the prevention and treatment of diabetic kidney injury by *R. nasutus* from a new perspective of “metabolism-oxidation coupling”.

## 2. Materials and Methods

### 2.1. Reagents and Chemicals

*R. nasutus* was obtained from the Yulin region in Guangxi province of China, where the climate is mild and humid. *R. nasutus* plant was authenticated by Professor Haixia Chen and the voucher specimen (No. TJC 2018001) was deposited at Laboratory of Natural Medicine, School of Pharmaceutical Science and Technology, Faculty of Medicine, Tianjin University. β-actin (CW0264M) was purchased from CWBIO (Jiangsu, China). The primary antibodies p-Akt (4060S), PI3K (4257S), and Akt (4691S) were obtained from Cell Signaling Technology (Danvers, MA, USA). The p-PI3K primary antibody (#13621) was sourced from Signalway Antibody LLC (Greenbelt, MD, USA). GLUT-4 (WL02425), mTOR (WL02477) and p-mTOR (Ser2448, WLH3897) were acquired from Wanleibio (Shenyang, China). Anti-rabbit IgG (H+L) (AB0171) and Anti-mouse IgG (H+L) (AB0172) were obtained from Abways (Shanghai, China).

### 2.2. Preparation of AE

The extraction manner was according to the earlier information with some modifications [12]. The dried aerial parts of *R. nasutus* were pulverized into a coarse powder. The powder was subjected to aqueous extraction using a solid-to-liquid ratio of 1:10 (*w*/*v*) with distilled water. The extraction was performed under reflux for 1 h, and this process was repeated three times. The combined extracts were concentrated under reduced pressure at 45 °C using a rotary evaporator (Yarong, China). After freeze–drying (Boyikang, Beijing, China), the resulting aqueous extract (AE) was stored at −20 °C until further use.

### 2.3. Serum Pharmacochemistry Analysis of AE

#### 2.3.1. Animals

Sprague Dawley (SD) rats, having a weight of 200 ± 10 g and aged 6 weeks, were obtained from Beijing Vital River Laboratory Animal Technology Co., Ltd. (license SCXK (Beijing, China) 2021-0011). All animal tests were carried out with the approval of the Experimental Animal Ethics Committee at the Institute of Radiation Medicine, Chinese Academy of Medical Sciences, Tianjin (Approval number: SYXK (Jin) 2019-0002). SD rats were randomly given to either an unmarked group or a medication management group, each consisting of six rats. The administration regimen was according to the study [13].

#### 2.3.2. Preparation of Serum Sample

The groundwork manner of serum samples was according to the earlier report [14]. Briefly, 800 μL of methanol (pre-frozen at −20 °C) was added to 200 μL of serum. After centrifuging, the supernatant liquid was dried with nitrogen. A total of 200 μL methanol (5%, *v*/*v*) was added into the sample and the sample was re-dissolved. The serum samples were obtained after centrifugation.

### 2.4. Q Exactive HF Orbitrap LC–MS/MS Analysis of AE and AEB

LC–MS/MS analysis was executed on a Q Exactive HF Orbitrap mass spectrometer integrated with an Ultimate 3000 RSLC nano system (Thermo Fisher Scientific, Waltham, MA, USA). The total components of AE and the composition of AE absorbed into blood (AEB) were analyzed according to a previous study [15]. LC–MSS/MS analysis was carried out according to the study of Li et al. with slight modification [16]. The mobile phase was composed of methanol (A) and water (B), with a flow rate put at 0.2 mL/min. The gradient program commenced at 5% B, gradually increasing to 60% B over 20 min, followed by a further increase to 98% B by the 28 min mark, which was then maintained for an additional 2 min. The scanning range of MS was 100–1200 Da.

### 2.5. Network Pharmacology Analysis

The Pubmed database (https://pubmed.ncbi.nlm.nih.gov/, access on 11 February 2026) for each compound to Smiles was used to choose AEB’s active ingredient further. Prediction of targets of potentially active compounds using the Swisstargetprediction database. “Homo Sapiens” was selected, and potential protein targets of AEB components were predicted with “Probability > 0” as the screening condition. Standardize the gene names of the targets using the Uniprot database (https://www.uniprot.org/, access on 11 February 2026).

Using Genecards (https://www.genecards.org/, access on 11 February 2026), TTD (https://db.idrblab.net/ttd//, access on 11 February 2026), OMIM (https://www.omim.org//, access on 11 February 2026), and DrugBank (https://go.drugbank.com//, access on 11 February 2026) databases to search for targets related to diabetic nephropathy with the keyword “Diabetic nephropathy”. The potential targets of AEB-related diabetes were obtained from Venny 2.1.0 (http://www.liuxiaoyuyuan.cn//, access on 11 February 2026). Then, AEB’s active compounds and anti-diabetic nephropathy targets were imported into Cytoscape 3.9.1 software to show the interaction network between AEB compounds and diabetes key targets. Protein interaction data were retrieved from STRING (https://www.string-db.org//, access on 11 February 2026). Cytoscape software was used to obtain and visualize high-confidence PPI networks to explore the relationship between AEB and diabetic nephropathy. The DAVID Functional Annotation Tool (available at https://davidbioinformatics.nih.gov/, access on 11 February 2026) was utilized to conduct Gene Ontology (GO) enrichment analysis and KEGG pathway analysis.

### 2.6. In Vitro Anti-Hypoglycemic Activity Analysis

The enzyme inhibitory activity of α-glucosidase could be explained as part of the mechanism of hypoglycemic activity. The method for assessing α-glycosidase inhibitory activity was based on our prior study [15]. Briefly, the p-nitrophenyl-α-D-glucopyranoside (pNPG) was used as the substrate. α-glucosidase (0.5 U/mL in 0.1 M phosphate buffer, pH 6.8) was mixed with varying concentrations of AE and incubated at 37 °C. Subsequently, 5 mM pNPG was added to initiate the reaction. After further incubation at 37 °C for 20 min, the reaction was terminated by adding 1 M Na_2_CO_3_. The absorbance was measured at 405 nm by microplate reader (Tecan, Männedorf, Switzerland). Acarbose was used as the positive control.

Furthermore, L6 myotubes were used to investigate the cytotoxicity and glucose uptake of AE. Standard cultivation of L6 rat skeletal myoblasts involved DMEM enriched with 10% FBS and 1% antibiotics. Following expansion to 80% density, cells were dissociated with 0.25% trypsin-EDTA for subculturing. To induce maturation, the growth medium was replaced with a 2% horse serum-DMEM solution once confluence was achieved. This differentiation phase lasted 6 days, with biennial medium exchange. Only morphologically confirmed, multinucleated myotubes were employed for subsequent experimental assays. The L6 myotube cells were treated with AE (0, 25, 50, 75, 100, 200, 400 μg/mL) for 24 h. Then, the cell viability was judged using CCK-8 (GlpBio, Montclair, CA, USA). The glucose uptake capacity was investigated via L6 myotube cells according to our earlier studies [17]. Briefly, the L6 myotube cells were treated with AE (0, 25, 50, 75 μg/mL) for 4 h. The glucose uptake capacity was quantified using the fluorescence intensity of 2-NBDG [18].

### 2.7. In Vitro Antioxidant Activity Analysis

The iron reduction antioxidant capacity (FRAP), DPPH and ABTS free radical scavenging assays are important methods for evaluating the in vitro antioxidant activity [15]. IC_50_ was used to evaluate the DPPH and ABTS radical scavenging ability of AE. The result of FRAP is expressed by absorbance value. Furthermore, the rat-derived small intestinal crypt epithelial cell line (IEC-6 cell) was utilized as an in vitro model in this study. To simulate intestinal epithelial injury, the cells were challenged with hydrogen peroxide (H_2_O_2_) to induce oxidative stress [18]. IEC-6 cells were treated with different concentrations of AE (0, 50, 100, 200, 400, 600 µg/mL) and H_2_O_2_ (0, 110, 120, 130, 140, 150, 160 μM) to assay the cell cytotoxicity. Then IEC-6 cells were added with optimal inhibitory concentration of H_2_O_2_ for 6 h, and AE was added to intervene for 24 h. In the same way, cell viability was judged using the MTT analysis.

### 2.8. In Vivo Protection Effects on Diabetic Nephropathy

#### 2.8.1. Establishment of the DN Model and Treatment

After a one-week acclimatization, SD rats were fasted for 12 h and then injected with streptozotocin (STZ, 50 mg/kg) to established DN model [19]. The rats were divided into five experimental groups, with 6 rats per group: I: Normal control group (Con); II: Model group (Mod); III: Diabetes rats treated with metformin (180 mg/kg, Met); IV and V: Diabetes rats treated with AE (250 mg/kg (AEL), 500 mg/kg (AEH)). This dosage was determined based on previous research reports [20]. The study has been approved by the Animal Ethical and Welfare Committee (SYXK (Jin) 2019-0002). Termination of the experiment was followed by euthanasia of all rodent subjects; thereafter, blood and kidney specimens were obtained for further research.

#### 2.8.2. In Vivo Antioxidant Activity Analysis

Renal pathology analysis was assessed using established methods [21]. The quantitative results of the pathology were analyzed by Image J 1.8.0.345. For Masson’s trichrome and PAS staining, the Color Deconvolution plugin was utilized to specifically isolate the blue-stained collagen fibers and magenta-stained glycoproteins, respectively. A standardized global threshold was applied to all images to identify positive staining areas. The results were expressed as the Area Fraction (%), calculated as the ratio of the positive-stained area to the total tissue area. Kidney samples were processed into a 1:9 (*w*/*v*) homogenate using chilled normal saline. Following thorough comminution in an ice-water bath, the mixture underwent centrifugation (12,000× *g*, 10 min, 4 °C) to isolate the clear supernatant. Then, superoxide dismutase (SOD), catalase (CAT),glutathione peroxidase (GSH-Px), and malondialdehyde (MDA) biochemical parameters were detected.

### 2.9. Western Blot

Western blot was used to study the effect of AE on PI3K/AKT signal pathway and GLUT4 in L6 cells and PI3K/AKT/mTOR signal pathway in kidney tissue. Cells were disturbed with RIPA lysis buffer. Western blot was performed according to the method of our previous studies [18]. Briefly, protein concentrations were quantified using a BCA assay kit and standardized accordingly. Subsequently, equal amounts of protein were resolved by SDS-PAGE and transferred onto PVDF membranes. The membranes were then subjected to overnight incubation with primary antibodies at 4 °C, followed by treatment with secondary antibodies for 1 h at room temperature. Target proteins, including PI3K, AKT, mTOR, and GLUT4, were detected using β-actin as an internal control. Band intensities were quantified via Chemi Analysis and further processed with ImageJ software.

### 2.10. Statistical Analysis

The data of consequences were stated as mean ± SD and analyzed with GraphPad Prism 5.0. The dissimilarity among many groups was inspected using one-way ANOVA. The dissimilarity was considered to be statistically significant at *p* < 0.05.

## 3. Results

### 3.1. Chemical Composition Analysis of AE

The yield of AE was 23.83 mg/g *R. nasutus*. According to the component content determination results, AE is rich in flavonoids, polyphenols, and triterpenes. The total flavonoid content is the highest, reaching 2.54 ± 0.02 mg AEs/g *R. nasutus*. Total polyphenol content follows closely behind, at 2.32 ± 0.04 mg AEs/g *R. nasutus*. Additionally, AE also contains total triterpenes (0.87 ± 0.02 mg AEs/g *R. nasutus*) and total sterols (0.13 ± 0.07 mg AEs/g *R. nasutus*) (Table 1). Aqueous solution extraction is more conducive to collecting polyphenols and flavonoids, which follows the principle of similar solubility [22]. Furthermore, Q Exactive HF LC–MS results identified multiple potentially present compounds (Table S1). The results recognized 38 compounds, which contained 12 phenolic acids (ferulic acid, caffeic acid and gentisic acid), and 5 flavonoids (isoflavones and hesperidin). Furthermore, AE contains sesquiterpenes, coumarin derivatives, cinnamic acid derivatives, etc. (Table 2). It was similar with the previous studies highlighting ferulic acid [23], caffeic acid [24,25] and flavonoids [26]. The presence of sesquiterpenes [27], coumarin derivatives [28,29] and cinnamic acid derivatives [30] further enriches the chemical diversity of *R. nasutus*.

### 3.2. Component Analysis of AEB

To explore the potential bioavailable constituents of AE, component changes absorbed into plasma were used to detect component metabolism. The AEB was analyzed using Q Exactive HF LC–MS. There are 23 prototypes and 2 metabolites in AEB (Table 2). Among them, caffeic acid, syringic acid, naringenin and salvianolic acid B were prototype compounds, while dihydroferulic acid and ferulic acid were discovered as metabolites. The potential metabolites compounds include 2-hydroxybenzaldehyde and 3-methoxy phenylacetic acid [31,32].

### 3.3. Network Pharmacological Studies

**Figure 1:**
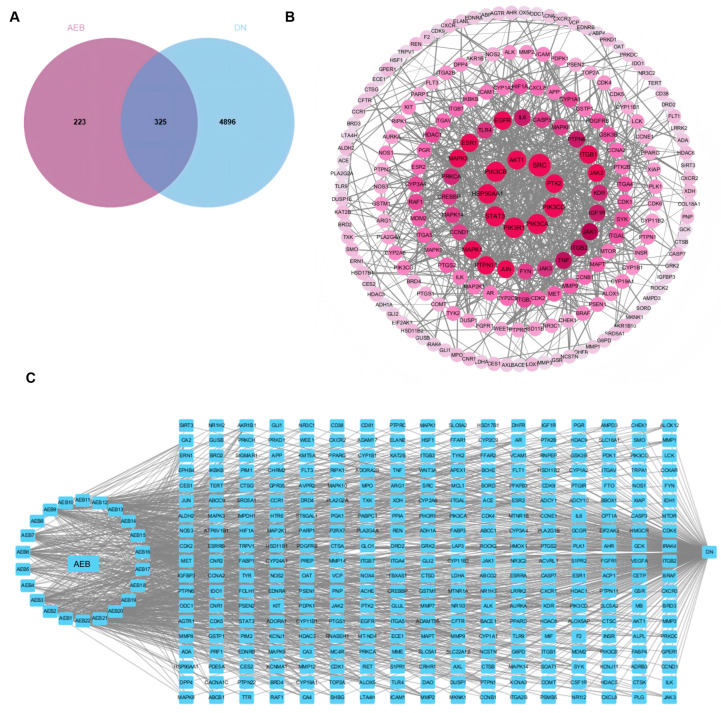
Network pharmacology results of AEB and DN. (**A**) Venn diagram; (**B**) PPI network among key targets; (**C**) The “disease-targets-components” associated with AEB and DN.

Network pharmacology is the multi-target and multi-component analytical framework analysis, which is consistent with the complexity of herbs and enables the identification of key drug targets and signal pathways involved in disease modulation [33]. The results show that there are 325 common target points between AEB and DN (Figure 1A). DN active compounds from AEB were screened to construct a “disease-compound-target” network. Cytoscape was used to show the “disease-compound-target” diagram (Figure 1C). It identified 22 compounds as key active ingredients because these values exceed their respective averages (Table S2). The main active compounds included esters, flavonoids, phenolic acids, terpenoids and naphthoquinones. AEB key targets were obtained by the PPI network (Figure 1B). A total of 325 core target genes were uploaded to the STRING. The remote target genes were removed, and the PPI network was obtained by covering with “high confidence > 0.900”. The PPI network held 325 nodes and 886 edges, with a standard nodule amount of 5.45. With all three screening values greater than the average value as the screening criteria, 41 target genes were obtained as the key targets. The top 10 key genes were SRC, PIK3R1, PIK3CA and others (Table S3).

**Figure 2:**
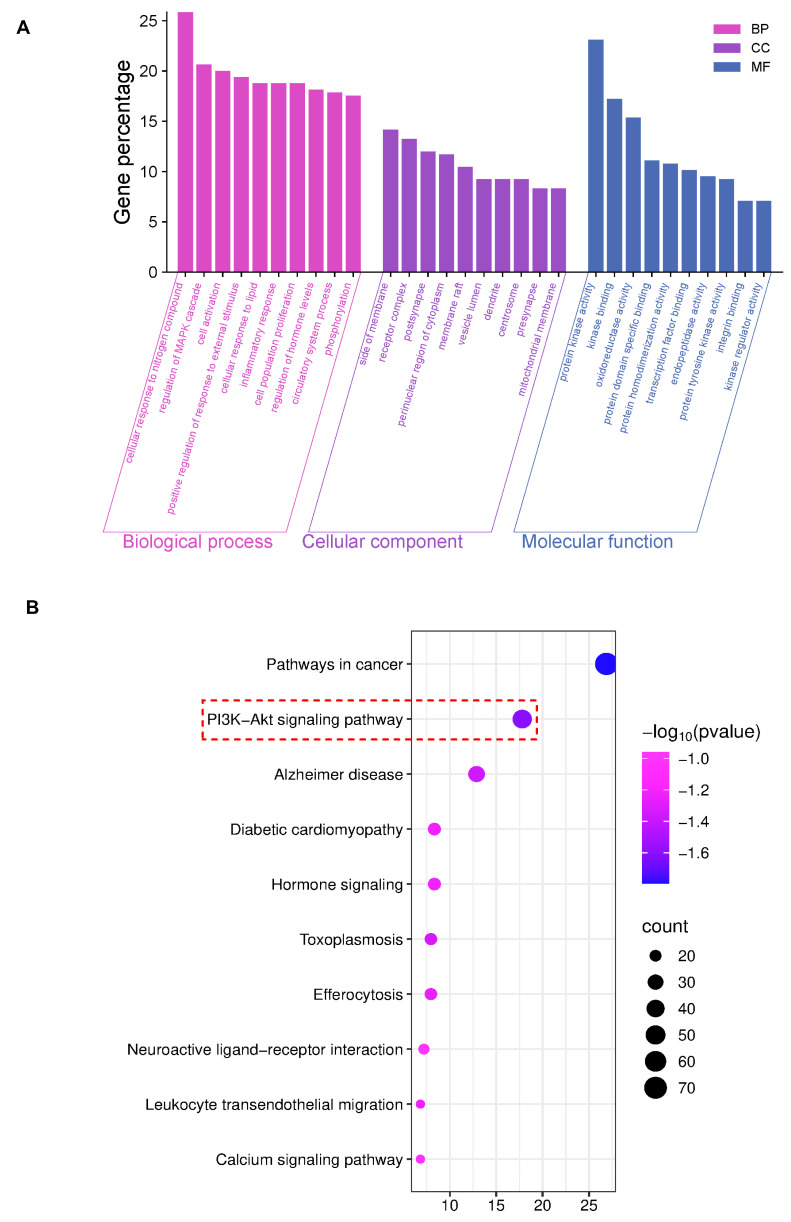
Network pharmacology results of AEB and DN. (**A**) The results of GO analysis; (**B**) The results of KEGG analysis (The key research pathway is highlighted by the red dashed box).

The GO analysis results showed the top 10 items (*p* < 0.01) for visual presentation (Figure 2A). And the KEGG improvement analysis recognized 264 pathways related to AE and DN. The results showed the top 10 genes were significantly enriched (*p* < 0.01) (Figure 2B). KEGG improvement analysis recognized several pathways, with particular importance on the PI3K/AKT signaling pathway. Based on the research reports, this study explored the PI3K/AKT signaling pathway in the following studies.

**Table 2:** Tentative identification of compounds in AEB by Q Exactive HF Orbitrap LC–MS/MS analysis.

No	Rt (min)	Name	Positive Ion or Negative Ion (m/z)			Element Composotion	Molecular Weight (Da)	MS/MS (m/z)	Source
			M±H	Indicated	ppm				
1	1.798	2,4-Dihydroxycinnamic acid	M-H	180.04176	−2.75	C_9_H_8_O_4_	179.03444	179[M-H]^−^, 161[M-H-H_2_O]^−^, 135[M-H-COO]^−^, 130[M-H-OH-2O]^−^, 117[M-H-COO-H_2_O]^−^	Prototypes
2	2.365	4-Acetyl-3-hydroxy-5-methylphenyl β-D-glucopyranoside	M-H	328.1149	−2.67	C_15_H_20_O_8_	327.10767	327[M-H]^−^,283[M-H-CH_3_-CHO]^−^,256[M-H-C_2_H_4_O-C_2_H_4_]^−^,248[M-H-C_2_H_5_O-2OH]^−^,232[M-H-C_2_H_5_O_2_-2OH]^−^,212[M-H-C_2_H_5_O_3_-2OH]^−^,192[M-H-C_2_H_5_O_2_-2OH-C_3_H_4_]^−^,165[M-H-C_2_H_5_O_2_-2OH-C_4_H_3_O]^−^,147[M-H-C_2_H_5_O_2_-2OH-C_4_H_3_O-H_2_O]^−^	Prototypes
3	5.298	1-O-(4-Coumaroyl)-beta-D-glucose	M-H	326.0922	4.52	C_15_H_18_O_8_	325.09192	325[M-H]^−^, 239[M-H-2CHO-CO]^−^, 231[M-H-C_6_H_6_O]^−^, 197[M-H-2CHO-CO-C_2_H_2_O]^−^, 179[M-H-2CHO-CO-C_2_H_2_O-H_2_O]^−^, 167[M-H-2CHO-CO-C_2_H_2_O-CH_3_OH]^−^	Prototypes
4	6.636	trans O-Coumaric acid	M+H	164.04690	−2.70	C_9_H_8_O_3_	165.05421	165[M+H]^+^, 147[M+H-H_2_O]^+^,	Prototypes
5	7.321	Homogentisic acid	M+H	168.04226	−2.30	C_8_H_8_O_4_	169.04901	169[M+H]^+^, 118[M+H-3OH]^+^	Prototypes
6	7.588	3, 5-Dihydroxybenzaldehyde	M-H	138.03126	−3.12	C_7_H_6_O_3_	137.02399	137[M-H]^−^, 121[M-H-O]^−^	Prototypes
7	8.094	p-Hydroxymandelic acid	M+H	168.04178	−2.83	C_8_H_8_O_4_	169.04906	169[M+H]^+^, 118[M+H-3OH]^+^	Metabolites
8	10.943	3,4’,5,6,7-Pentamethoxyflavone	M+H	372.11775	−3.71	C_20_H_20_O_7_	373.12680	373[M+H]^+^, 344[M+H-CHO]^+^, 295[M+H-C_2_H_6_O_3_]^+^, 279[M+H-C_2_H_6_O_4_]^+^, 269[M+H-C_2_H_6_O_3_-2CH]^+^, 209[M+H-CHO-C_8_H_7_O_2_]^+^, 193[M+H-C_2_H_6_O_3_-2CH-C_6_H_4_]^+^, 149[M+H-C_2_H_6_O_3_-2CH-C_6_H_4_-C_2_H_4_O]^+^, 118[M+H-C_2_H_6_O_4_-C_11_H_13_O]^+^,	Prototypes
9	11.647	m-Coumaric acid	M+H	164.04689	−2.76	C_9_H_8_O_3_	165.05417	165[M+H]^+^, 147[M+H-H_2_O]^+^	Prototypes
10	12.873	3-Methoxyphenylacetic acid	M-H	166.0625	−3.0	C_9_H_10_O_3_	165.05516	165[M-H]^−^, 121[M-H-COO]^−^	Metabolites
11	13.209	Sinensetin	M+H	372.11987	−3.71	C_20_H_20_O_7_	373.1201	373[M+H]^+^, 353[M+H-CH_2_]^+^, 279[M+H-CH_2_-C_2_H_8_O_3_]^+^, 223[M+H-C_9_H_10_O_2_]^+^, 209[M+H-C_9_H_8_O_3_]^+^, 163[M+H-C_9_H_10_O_2_-2CO]^+^, 149[M+H-CH_2_-C_2_H_8_O_3_-C_9_H_6_O]^+^	Prototypes
12	13.526	Isosinensetin	M+H	372.11952	−3.71	C_20_H_20_O_7_	373.12686	373[M+H]^+^, 355[M+H-H_2_O]^+^, 279[M+H-H_2_O-C_3_H_8_O2]^+^, 270[M+H-C_4_H_7_O_3_]^+^, 149[M+H-C_4_H_7_O_3_-C_8_H_9_O]^+^, 118[M+H-H_2_O-C_3_H_8_O_2_-C_10_H_9_O_2_]^+^	Prototypes
13	17.428	Salvigenin	M-H	328.09361	−3.28	C_18_H_16_O_6_	327.08633	327[M-H]^−^, 243[M-H-C_5_H_8_O]^−^, 195[M-H-C_5_H_8_O_4_]^−^, 182[M-H-C_5_H_8_O_4_-CH]^−^, 153[M-H-C_5_H_8_O_4_-CH-CHO]^−^, 147[M-H-C_9_H_8_O_4_]^−^, 116[M-H-C_5_H_8_O_4_-CH-CHO-C_3_H_2_]^−^	Prototypes
14	21.897	Ethyl 3-(3,4-dihydroxyphenyl)propionate	M-H	210.08858	−3.01	C_11_H_14_O_4_	209.08128	209[M-H]^−^, 182[M-H-C_2_H_5_]^−^, 112[M-H-C_2_H_5_-C_3_H_2_O_2_]^−^	Prototypes
15	23.354	2β,9α-Diacetoxy-trans-decalin	M+H	254.1509	−3.66	C_14_H_22_O_4_	255.15799	255[M+H]^+^, 237[M+H-H_2_O]^+^, 226[M+H-C_2_H_5_]^+^, 180[M+H-CH_3_COO-O]^+^, 149[M+H-C_3_H_8_-COO-H_2_O]^+^	Prototypes
16	24.937	Embelin	M-H	294.1823	−2.77	C_17_H_26_O_4_	293.17502	293[M-H]^−^, 265[M-H-CO]^−^, 182[M-H-C_2_H_3_-3CO]^−^, 112[M-H-3CO-C_6_H_11_O]^−^	Prototypes
17	24.975	5-Hydroxy-1-(4-hydroxy-3-methoxy-cyclohexyl)decan-3-one	M+H	294.1820	−3.69	C_17_H_26_O_4_	295.18930	295[M+H]^+^, 249[M+H-CH_3_O-H_2_O]^+^, 244[M+H-CH_3_-2H_2_O]^+^, 227[M+H-CH_3_-2H_2_O-OH]^+^, 149[M+H-C_6_H_6_O_2_-2H_2_O]^+^, 118[M+H-C_9_H_17_O-2H_2_O]^+^	Prototypes
18	25.255	3-(2,2,5,6-Tetramethyl-5-(((2-oxo-2H-chromen-7-yl)oxy)methyl)-1-oxaspiro [2.5]octan-4-yl)propanoic acid	M+H	414.2026	−3.89	C_24_H_30_O_6_	415.20990	415[M+H]^+^, 318[M+H-C_4_H_5_-COO]^+^, 274[M+H-C_4_H_5_-2COO]^+^	Prototypes
19	25.954	Sterebins A	M+H	310.2132	−3.92	C_18_H_30_O_4_	311.22034	311[M+H]^+^, 293[M+H-H_2_O]^+^, 274[M+H-H_3_O_2_]^+^, 230[M+H-H_3_O_2_-H_2_O_2_]^+^	Prototypes
20	26.199	Rhinacanthone	M+H	242.09349	−3.31	C_15_H_14_O_3_	243.10078	243[M+H]^+^, 209[M+H-2OH]^+^, 192[M+H-3OH]^+^, 163[M+H-3OH-C_2_H_5_]^+^, 149[M+H-3OH-C_2_H_5_-CH_2_]^+^,	Prototypes
21	26.719	[6]-Gingerdiol	M-H	296.1977	−3.44	C_17_H_28_O_4_	295.17999	295[M-H]^−^,277[M-H-H_2_O]^−^,233[M-H-C_2_H_5_OH]^−^,182[M-H-C_2_H_5_OH-CH_3_-2H_2_O]^−^,115[M-H-C_2_H_5_OH-C_6_H_10_-2H_2_O]^−^	Prototypes
22	26.792	Phenylpyruvic Acid	M+H	164.04734	−3.03	C_9_H_8_O_3_	165.05412	165[M+H]^+^, 149[M+H-O]^+^, 118[M+H-CH_3_O_2_]^+^	Prototypes
23	27.116	Thujopsenic acid	M+H	220.1456	−3.24	C_14_H_20_O_2_	221.15291	221[M+H]^+^, 149[M+H-C_3_H_8_-CO]^+^, 118M+H-C_3_H_8_-CH_3_O-CO]^+^	Prototypes
24	27.147	(2-Methyl-heptyl)-malonic acid diethyl ester	M-H	272.1979	−2.99	C_15_H_28_O_4_	271.19064	271[M-H]^−^, 239[M-H-2O]^−^, 182[M-H-C_2_H_5_O-COO]^−^, 115[M-H-C_2_H_5_O-C2H5-COO-COOH]^−^	Prototypes
25	27.993	2,6-Di-tert-butyl-1,4-benzoquinone	M+H	220.1456	−3.32	C_14_H_20_O_2_	221.15399	221[M+H]^+^, 182[M+H-C_3_H_3_]^+^, 115[M+H-2H_2_O-C_5_H_10_]^+^	Prototypes

### 3.4. In Vitro Glucose-Lowering Potential

**Figure 3:**
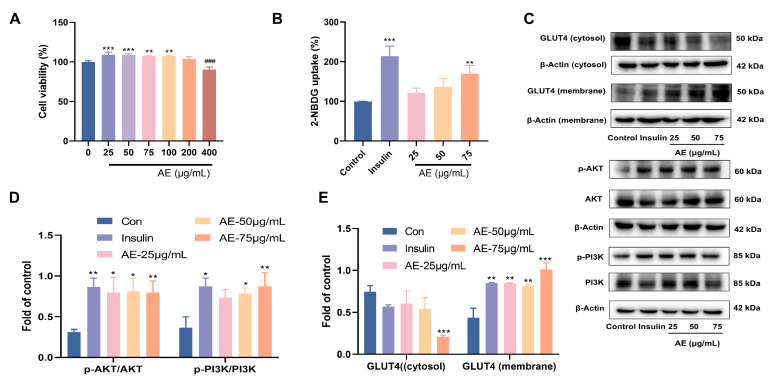
Effects of glucose uptake activity of AE; (**A**) Cytotoxicity of AE on L6 cells; (**B**) The results of 2-NBDG uptake; (**C**–**E**) Expression of AE on GLUT4 and PI3K/Akt. (**A**–**E**): Compared with the control group, *, *p* < 0.05; **, *p* < 0.01 and ***, *p* < 0.001; ^###^, *p* < 0.001.

The inhibition of α-glucosidase plays a crucial role in the anti-hypoglycemic activity [34]. The results proved that AE showed α-glucosidase inhibitory activity, with an IC_50_ value of 77.33 ± 0.01 μg/mL (Table 1). Glucose uptake is a key step in improving blood sugar levels. Facilitating glucose uptake and utilization in peripheral tissues (e.g., muscle and fat) influences the reduction of blood glucose concentration [35]. First of all, the results of cytotoxicity of AE indicated that treatment with different concentrations of AE (25–400 μg/mL) did not show significant cytotoxicity on L6 myotubes (Figure 3A). Based on these safety profiles, lower concentrations of 25, 50, and 75 µg/mL were selected for subsequent bioactivity and mechanistic investigations. The results of glucose uptake showed significance at 75 μg/mL with a value of 131.12% (*p* < 0.01) (Figure 3B). When AE concentration was 75 μg/mL, the results indicated the important glucose uptake ability of 131.12% (*p* < 0.01) (Figure 3B).

Furthermore, this study assayed the expression of PI3K and AKT proteins under the treatment of AE in L6 myocytes according to the results of network pharmacology. The AE treatment increased the proportion of phosphor-AKT to total AKT in L6 myocytes when compared to control group. Similarly, the proportion of phospho-PI3K to total PI3K was raised (Figure 3C,D). GLUT4 is known to be pivotal in facilitating early glucose uptake [36]. AE treatment promoted the redistribution of GLUT4, increasing its presence in the plasma membrane relative to the cytosol. The phenomenon was similar to that of insulin (Figure 3C,E). The PI3K/AKT phosphorylation pathway plays a critical role in the transposition of GLUT4 in L6 myotubes. The results showed that AE might enhancement of glucose uptake by PI3K/AKT and GLUT4 signal pathways [37].

### 3.5. Evaluation of the Antioxidant Activity of AE

As oxidative stress represents the final common pathway and central driver of hyperglycemia-induced renal damage, potent antioxidant intervention is indispensable to neutralize reactive oxygen species and arrest the progressive transition from metabolic dysfunction to irreversible renal failure [38]. AE showed an important DPPH and ABTS scavenging capabilities (IC_50_ value of 8.78 ± 0.97 μg/mL, 23.62 ± 0.13 μg/mL) (Table 1). FRAP results showed that when AE concentration was 104.17 μg/mL, the absorbance value was 0.112. When the concentration of ascorbic acid is 4.17 μg/mL, the absorbance value is 0.237 (Table 1). Compared with previously reported compounds with antioxidant activity, AE showed potential iron reduction ability [39]. It could be concluded that *R. nasutus* has promising antioxidant activity.

**Figure 4:**
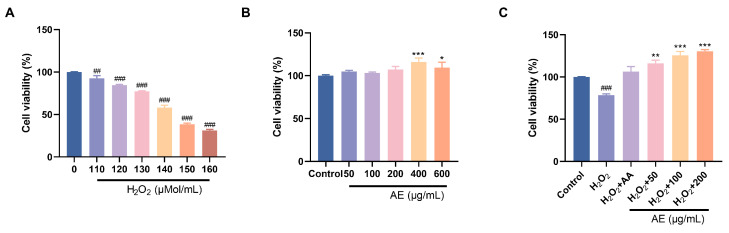
Effects of antioxidant activity of AE. (**A**,**B**) Cell viability of H_2_O_2_ and AE; (**C**) Effects of AE on H_2_O_2_-induced IEC-6 cells. A, B: Compared with the control group, *, *p* < 0.05; **, *p* < 0.01 and ***, *p* < 0.001; ^##^, *p* < 0.01, ^###^, *p* < 0.001.

In the treatment with oral drugs, reducing intestinal oxidative damage is of utmost importance [40]. Persistent hyperglycemia can disrupt the intestinal mucosal barrier, leading to the induction of oxidative stress and systemic inflammation. By using drugs or functional components to eliminate reactive oxygen species in the intestine and enhance antioxidant defense, it is possible to protect the integrity of the intestinal mucosa and ease the oxidative destruction. Therefore, IEC-6 cells were used to investigate the ability of AE to reduce oxidative damage. Within the 50–600 μg/mL series, AE indicated no cytotoxic effects on IEC-6 cells. Instead, it exhibited a certain promotion effect (Figure 4B). Excessive H_2_O_2_ penetration into cells induces oxidative stress, potentially leading to apoptosis. Alleviating H_2_O_2_-induced oxidative damage is a manifestation of anti-oxidation and alleviating oxidative damage. IEC-6 cell cytotoxicity results showed that exposure to increased H_2_O_2_ concentrations (110, 120, 130, 140, 150, 160 μM) for 6 h decreased cell viability from 90% to 40% (Figure 4A). This showed the poisonous effects of H_2_O_2_ on IEC-6 cells under conditions that simulate oxidative stress. H_2_O_2_ (130 μM) could reduce cell viability to around 75%. It indicated the induction of oxidative damage of IEC-6 cells. Furthermore, AE showed important protecting belongings on H_2_O_2_-induced destruction in IEC-6 cells (Figure 4C). Compared to ascorbic acid, AE exhibited a notable increase in cell viability.

### 3.6. In Vivo Protection Effects on Diabetic Nephropathy

**Figure 5:**
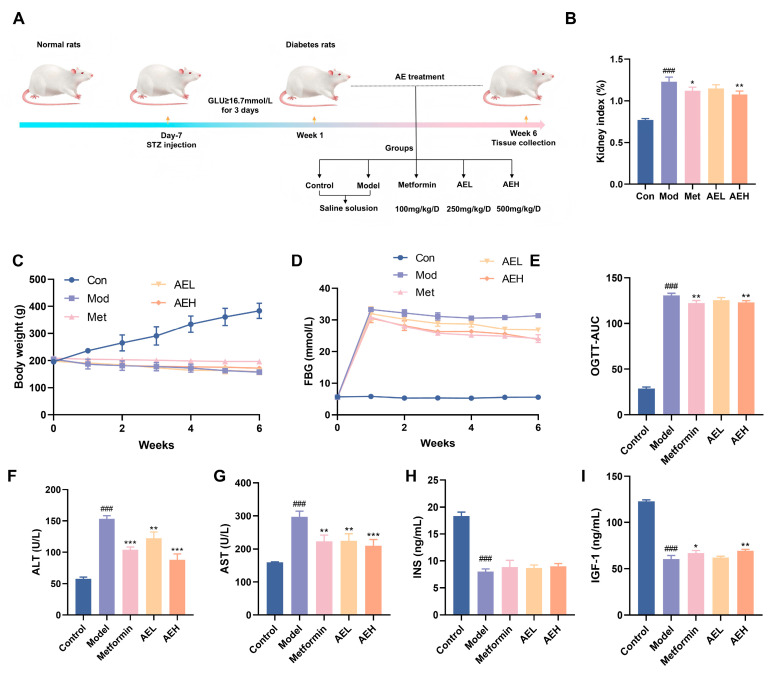
Effects of AE on DN rats. (**A**) Schematic representation of the experimental. (**B**) Kidney index levels. (**C**) Weight change in rats. (**D**) Fasting blood glucose (FBG) levels in rats. (**E**) The results of oral glucose tolerance. (**F**–**I**) ALT, AST, INS and IGF-1 levels in each group. All measurements were run in triplicate (*n* = 6). Compared with the control group, ^###^, *p* < 0.001. Contrast with the model group, *, *p* < 0.05; **, *p* < 0.01 and ***, *p* < 0.001.

To assess the beneficial possible of *R. nasutus* in DN, STZ-induced DN model was accepted (Figure 5A). The consequence indicated that the body weight of STZ-induced rats stayed stale or dropped over the 6-week experimental time, in contrast to the constant weight acquire watched in the control group. Neither AE nor metformin (Met) treatment reversed this weight alteration (Figure 5C). Furthermore, rats in the model group showed elevated lifeblood glucose position. Although both Met and AE interventions moderated hyperglycemia, blood glucose concentrations remained elevated (Figure 5D). Oral glucose tolerance test (OGTT) results proved that AE mediation lessened in DN rats, an effect comparable to that of Met administration (Figure 5E). This suggested that AE could improve glucose tolerance under diabetic conditions. Furthermore, the kidney index was markedly larger in DN rats, while both Met and high-dose AE (AEH) treatments effectively suppressed this increase. It indicated that AEH partially ameliorates diabetes-associated renal hypertrophy.

Serum position of ALT and AST were exalted in model group compared with control group (ALT: 153.18 ± 5.00 U/L vs. 57.51 ± 3.03 U/L; AST: 296.85 ± 18.03 U/L vs. 159.24 ± 1.22 U/L), confirming STZ-induced hepatotoxicity in addition to the diabetic state. In contrast, both AEL and AEH groups, as well as the Met group, exhibited significantly reduced ALT and AST levels (Figure 5F,G). These results showed AE could attenuate STZ-induced liver injury in a manner similar to metformin [41]. In DN, hepatic function indirectly exacerbates the progression of renal injury through multiple pathways, including the regulation of drug metabolism, toxin clearance, and systemic inflammatory responses [42]. The observed alleviation of liver injury by AE in diabetic rats implicates its potential to attenuate subsequent renal functional impairment. Furthermore, serum position of insulin (INS) was very much reduced in the model group, similar with insulin-like growth factor-1 (IGF-1) (Figure 5H,I). It was suggested that STZ induction not only impairs pancreatic function but may also compromise the hepatic synthesis of IGF-1. Treatment with AE resulted in a partial restoration of both insulin and IGF-1 levels, an effect comparable to that of Met, showing a beneficial modulatory role of AE on the abnormal discharge of insulin and related factors in DN rats.

### 3.7. Renal Histopathological Assessment

**Figure 6:**
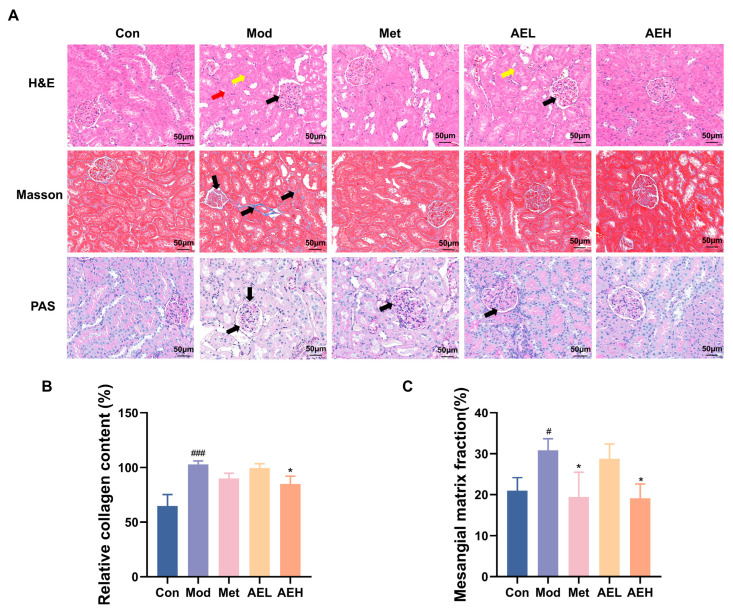
(**A**) Representative renal photomicrographs with staining. (**B**) Quantitative analysis of renal collagen fiber content based on Masson’s trichrome staining. The black arrow highlights glomerular matrix expansion and basement membrane thickening. (**C**) Statistical analysis of the glomerular basement membrane thickness to glomerular area ratio from PAS staining. Compared with the control group, ^#^, *p* < 0.05, ^###^, *p* < 0.001. Compared with the model group, *, *p* < 0.05.

H&E results of kidney tissues from the control group revealed intact glomerular architecture with normal size and cellularity, without evidence of mesangial matrix expansion, basement membrane thickening, or tubular injury (Figure 6A). In contrast, renal sections from the model group showed serious pathological change, containing noticeable thickening of the glomerular cellar layer, expansion of the mesangial matrix, necrosis of tubular epithelial cells, and prominent vacuolar degeneration. Treatment with both AEH and Met resulted in a noticeable amelioration of these injuries, distinguished by only faint mesangial matrix hyperplasia and the important decrease in the strictness of tubular vacuolar deterioration contrasted to the model group.

Masson’s trichrome staining was used to judge collagen sworn statement, a key sign of renal fibrosis. The results showed that collagen fibers were stained blue, cell nuclei were stained blue-black, and muscle fibers were stained red (Figure 6A,B). The kidney interstitium of model group showed a large, depressed collagen thread sworn statement around glomeruli and renal tubules. It showed the development of fibrosis. This aberrant collagen accumulation was substantially reduced in the AEH-treated group.

Furthermore, Periodic acid–Schiff (PAS) staining confirmed the histopathological findings (Figure 6A,C). A significant increase in the glomerular basement membrane thickness and an elevated ratio of glomerular basement membrane to glomerular area were observed in the model group. Diabetic renal injury was confirmed. However, mediation with AE and Met effectively slender the thickening of glomerular cellar layer. It was steady with the observations from H&E staining.

### 3.8. AE Administration Mitigates Renal Oxidative Stress in Diabetic Rats

**Figure 7:**
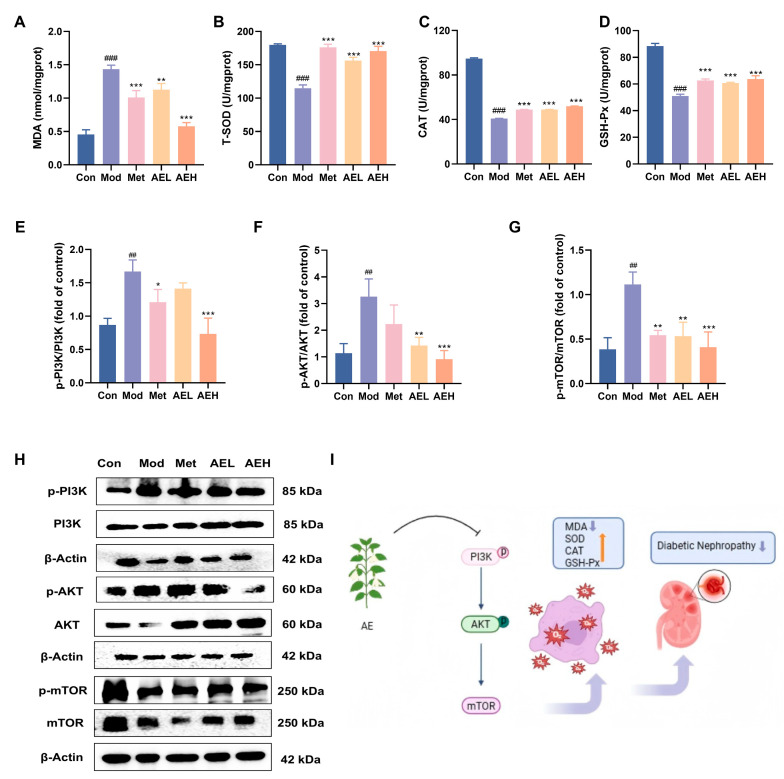
AE improves renal injury and oxidative emphasis in STZ-induced DN rats. (**A**–**D**) Renal levels of biochemical analysis. (**E**–**H**) Western blot analysis of AE. (**I**) Schematic representation of the putative mechanism by which AE ameliorates DN (An upward arrow denotes positive regulation, and a downward arrow negative regulation). Compared with the control group, ^##^, *p* < 0.01, ^###^, *p* < 0.001. Compared with the model group, *, *p* < 0.05; **, *p* < 0.01 and ***, *p* < 0.001.

To judge the effect of AE on the renal oxidative stress position in DN rats, key oxidative stress-related parameters were measured in renal tissues [43]. Compared with control group, the results in the model group showed drop in T-SOD (from 179.78 to 114.93 U/mgprot), CAT (from 94.77 to 40.84 U/mgprot) and GSH-Px’ (from 88.46 to 50.99 U/mgprot) activities. It was linked to the noticeable raise in MDA content (from 0.46 to 1.44 nmol/mgprot), indicating a state of pronounced oxidative stress in DN. Intervention with AE significantly reversed these alterations, leading to a notable increase in the action of all three enzymes and a simultaneous decrease in MDA position compared to model group. In the high-dose group (AEH), MDA levels were markedly reduced to 0.58 nmol/mgprot, effectively suppressing lipid peroxidation. Simultaneously, the activities of T-SOD, CAT, and GSH-Px’ were restored to 170.57 U/mgprot, 51.93 U/gprot, and 63.68 U/mgprot, respectively (Figure 7A–D).

### 3.9. Regulatory Effect of AE on the Signaling Pathways

The western blot results showed that the ratios of p-PI3K/PI3K, p-AKT/AKT and p-mTOR/mTOR in the renal tissues of the model group were increased. In contrast, these ratios were decreased in rats treated with AE or metformin (Figure 7E–H). These results recommend that AE could correlate with the PI3K/AKT/mTOR signaling pathway in the DN rats [44], which can alleviate kidney destruction (Figure 7I). The results were in agreement with the prediction results of network pharmacology analysis.

## 4. Discussion

In traditional medicine research, the integration of LC–MS analysis, network pharmacology, and serum pharmacochemistry is critically important. Serum pharmacochemistry analysis allows for the screening of active compounds that truly circulate in the blood, revealing potential pharmacologically active substances [45]. Network pharmacology constructs a multidimensional, systematically elucidating the mechanisms of multi-component synergy within disease networks [46]. The combination of these three approaches forms a broad research system: from chemical substance discovery and confirmation of in vivo vulnerability to clarification of pharmacological mechanisms. It can supply a technological foundation for explaining the substance arrangement and workings of action of traditional medicines or food. This integration significantly enhances the systematic rigor and credibility of medical research. *R. nasutus* is a medicinal plant that is commonly used, but there is little research on the constituents and action mechanism, especially on the aqueous extract. The results of LC–MS analysis for AE establishes a foundational dataset for research on the complex chemical composition of *R. nasutus*. Furthermore, the serum pharmacochemistry showed 23 components were detected. These components are highly likely to be the potential bioavailable constituents in the treatment of diabetes. While these components were successfully detected in plasma, their precise pharmacological contribution and pharmacokinetic profiles warrant further investigation in future quantitative studies. Caffeic acid derivatives contain phenylacetyl caffeic acid and caffeic acid ester. They could lower blood sugar and increase antioxidant markers [47]. Naringenin could reduce oxidative stress and necroptosis, apoptosis, and pyroptosis in random pattern skin flaps by enhancing autophagy [48,49]. Furthermore, salvianolic acid B has been demonstrated to have the capacity to protect against myocardial ischemia–reperfusion injury in diabetic rats [50]. Therefore, the potential anti-hypoglycemic and antioxidant activities of *R. nasutus* might be due to these phenolic acids or flavonoid components, which need further investigation. Network pharmacology analysis selected the PI3K/AKT pathway for in-depth study because it is well-accepted in its group with oxidative stress and DN, which are complicated in the upkeep of cellular functions such as apoptosis, inflammation, and metabolism [51]. Meanwhile, the degree of PI3K activation has an impact on glucose metabolism. The low-level expression of PI3K is often considered a major cause of elevated serum glucose concentrations. Moreover, the PI3K/AKT signaling pathway, as a key molecular hub regulating redox homeostasis, plays a “master control switch” role in combating oxidative stress and related tissue damage by strengthening the endogenous antioxidant defense mechanism [52]. Hence, applying LC–MS technology and network pharmacology analysis to study the active components entering the bloodstream can more deeply and accurately identify the bioactive components of *R. nasutus*.

Maintaining blood glucose levels is the primary prerequisite for intervening in the progression of DN. The results show that AE not only inhibits α-glucosidase activity [53] but also enhances the glucose uptake capacity of skeletal muscle L6 cells [54]. Previous studies have reported that caffeic acid and naringenin have significant abilities to enhance glucose uptake. This might be the reason why AE has glucose uptake capabilities [55,56]. Since skeletal muscle is the primary site for postprandial glucose disposal, the observed enhancement of glucose uptake in L6 cells provides a mechanistic basis for AE’s potential to reduce systemic “glucotoxicity.” The “glucotoxicity” induced by a high-glucose microenvironment is a critical trigger for the downstream oxidative stress cascade [57]. The results demonstrate that AE enhances the glucose uptake capacity of cells, which suggests a potential to mitigate excessive metabolic flux at the cellular source. Such regulation of glucose distribution might provide a prerequisite for attenuating the overproduction of mitochondrial reactive oxygen species (ROS) driven by high-glucose loading [58]. While these findings are currently limited to in vitro observations, the enhanced glucose clearance from the extracellular medium provides a preliminary mechanistic basis for future investigations into the antioxidant potential of AE within the pathological context of DN.

Meanwhile, antioxidant activities, such as free radical scavenging, work for the first stroke of protection against oxidative ability [59]. At a deeper cellular level, these activities play a beneficial role in alleviating intestinal oxidative stress, indicating that *R. nasutus* has the potential to protect intestinal barrier function. Maybe it was due to the antioxidative components such as phenolic acids and flavonoids. It has been noted that phenolic acid such as caffeic acid could protect against oxidative damage [60,61].

In DN rat models, mitigating oxidative stress is crucial for halting disease progression [62]. The biochemical analysis in SD rats demonstrated that AE administration significantly normalized abnormal metabolic indicators. This therapeutic efficacy could be attributed to the phenolic acid components identified in AE, which are recognized for their ability to modulate glucose uptake pathways. Specifically, AE treatment appears to stabilize blood glucose levels and improve glucose tolerance, thereby potentially alleviating liver dysfunction. The improvement in hepatic enzymes (ALT and AST) indicates that AE might alleviate systemic glucotoxicity by restoring liver metabolic homeostasis. Given that the liver is a central organ for glucose disposal, reducing hepatic oxidative damage could indirectly diminish the chronic metabolic burden on the kidneys, thereby contributing to the overall amelioration of diabetic nephropathy. Notably, caffeic acid is a compound with documented efficacy against diabetes and its complications [63], which was detected in the plasma following AE administration. It was noteworthy that this circulating component might be a key contributor to the observed therapeutic effects of AE. Beyond its metabolic role, caffeic acid, naringenin and other components like naringenin possess intrinsic anti-fibrotic and antioxidant properties, providing a biochemical link between oral intake and organ-specific protection [56,64,65]. Histopathological evaluations further confirmed that AE treatment confers a protective effect against STZ-induced diabetic kidney injury [66]. This renal protection is likely driven by the upregulation of key antioxidant enzymes and the suppression of lipid peroxidation, which collectively fortify the renal antioxidant defense system [67]. Hence, *R. nasutus* exhibits significant potential in preventing oxidative stress-mediated tissue destruction in DN.

**Figure 8:**
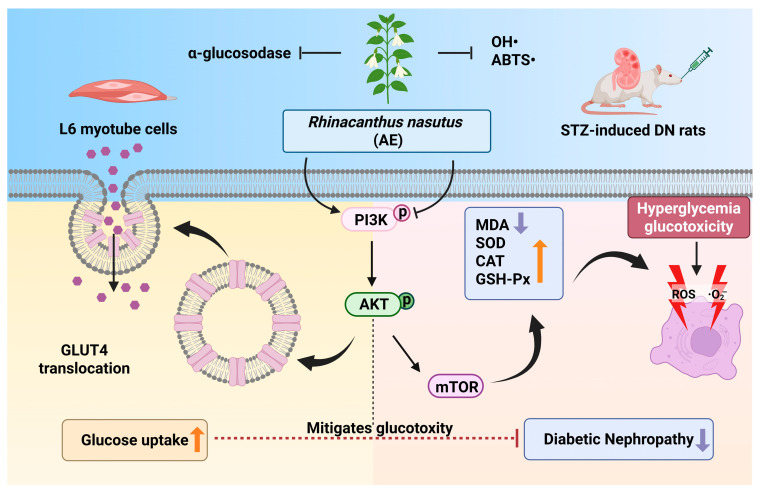
The potential mechanism of AE in DN (An upward arrow denotes positive regulation, and a downward arrow negative regulation). (Created in BioRender. Xudong, Y. (2025) https://BioRender.com/u5lb798, accessed on 31 December 2025).

The primary findings of this study stem from the integration of Q Exactive HF Orbitrap LC–MS/MS and serum pharmacochemistry, which identified 38 constituents in the AE and pinpointed 25 potential bioavailable metabolites (e.g., caffeic acid and naringenin). These results provide the evidence for the pharmacological basis of this botanical intervention. Mechanistically, we suggested that AE could enhance glucose uptake via the PI3K/AKT and GLUT4 pathway to promote glucose uptake and modulate the PI3K/AKT/mTOR signaling pathway to restore the antioxidant defense system, thereby breaking the ‘hyperglycemia-oxidative stress’ vicious cycle (Figure 8). Unlike previous studies focused on crude extracts, this study offers a multi-target mechanistic perspective, providing critical theoretical support for the clinical translation of *R. nasutus* into modern anti-diabetic therapeutics. While the modulation of the PI3K/AKT pathway aligns with our initial hypothesis, it is important to consider that the therapeutic effects of *R. nasutus* may be multifaceted. Given the diverse phytochemical profile of AE, other pathways, such as those involving anti-inflammatory responses or alternative glucose transporters, might also contribute to the observed renal protection [68]. These aspects warrant further investigation in future studies to fully elucidate the underlying mechanisms.

Despite the valuable insights provided by this study, several limitations warrant consideration for a comprehensive interpretation of the findings. Firstly, certain methodological limitations should be acknowledged. While our phytochemical profiling identified the potential constituents of AE, absolute quantification of marker compounds was not performed. To ensure experimental consistency, a standardized extraction protocol was strictly followed using a single batch of authenticated raw materials. However, establishing a comprehensive quality control strategy, including batch-to-batch reproducibility and marker-based normalization, remains an essential goal for our future pharmacological investigations. The network pharmacology analysis, while a powerful predictive tool, is inherently constrained by the availability of database information and should be interpreted as a preliminary hypothesis rather than definitive proof of mechanism. The antioxidant assays employed are primarily chemical-based and may not entirely reflect the complex, dynamic redox homeostasis within biological systems. While the STZ model effectively simulates hyperglycemia, it may not fully capture the multifactorial pathogenesis of human DN. Limitations such as the lack of human clinical data and the need to clarify synergistic effects among bioactive components remain, warranting further investigation for future clinical applications. Furthermore, while significant modulations in pathway proteins were observed, we acknowledge the absence of pharmacological inhibitors or genetic knockdown methods for loss-of-function validation, which constitutes a limitation of the present study. Nevertheless, previous studies in analogous models have demonstrated that the protective efficacy of similar bioactive compounds was markedly attenuated upon the administration of PI3K inhibitors [44,69]. These external findings could provide some indirect support for our findings. Consequently, further studies employing pathway-blocking experiments are warranted to definitively establish the causal link between AE, the PI3K/AKT/mTOR signal pathway, and the progression of DN.

## 5. Conclusions

In summary, this study primarily explored the reno-protective potential and associated mechanisms of *Rhinacanthus nasutus* aqueous extract (AE). The findings suggest that *R. nasutus* aqueous extract (AE) potentially ameliorates DN via a pivotal metabolic–oxidative coupling mechanism. By integrating phytochemical profiling with serum pharmacochemistry, 25 bioavailable metabolites were identified as the putative pharmacological basis. Mechanistically, AE treatment was associated with the modulation of the PI3K/AKT signaling pathway, which facilitates GLUT4-mediated glucose transport and may subsequently mitigate the metabolic drivers of oxidative stress. Concurrently, AE could reduce MDA and restore T-SOD, CAT, and GSH-Px, which fortifies the renal antioxidant defense system, likely mediated by the PI3K/AKT/mTOR axis in vivo. These results provide a scientific rationale for considering *R. nasutus* as a promising multi-target natural candidate for the management of DN. The action targets and clinical studies are needed for further validation.

## Figures and Tables

**Table 1:** Phytochemical composition and in vitro activity of AE.

Phytochemicals Composition	In Vitro Antioxidant Activities of AE			Enzyme Inhibitory Activity	
Total polyphenols (mg AEs/g *R. nasutus*)	2.32 ± 0.04	DPPH scavenging IC_50_ (μg/mL)	8.78 ± 0.97	α-Glucosidase inhibition IC_50_ (μg/mL)	77.33 ± 0.01
Total flavonoids (mg AEs/g *R. nasutus*)	2.54 ± 0.02	ABTS scavenging IC_50_ (μg/mL)	23.62 ± 0.13		
Total triterpenes (mg AEs/g *R. nasutus*)	0.87 ± 0.02	FRAP (A700, 104.17 μg/mL)	0.112 ± 0.01		
Total steroids (mg AEs/g *R. nasutus*)	0.13 ± 0.07				

## Data Availability

The original contributions presented in this study are included in the article/Supplementary Materials. Further inquiries can be directed to the corresponding author.

## Supplementary Materials

The following supporting information can be downloaded at: https://www.mdpi.com/article/10.3390/antiox15020252/s1, Table S1: Tentative identification of compounds in AE by Q Exactive HF LC–MS; Table S2: The key active ingredients of AEB associated with diabetic kidney disease; Table S3: The key targets of AEB associated with diabetic kidney disease (Top 10).

## Abbreviations

The following abbreviations are used in this manuscript:

ABTS	2,2′-Azino-bis-(3-ethylbenzothiazoline-6-sulphonate)
AKT	Protein kinase B
AKT1	AKT serine/threonine kinase 1
CCK-8	Cell counting kit-8
DPPH	1,1-Diphenyl-2-picrylhydrazyl radical 2,2-diphenyl-1-(2,4,6-trinitrophenyl) hydrazyl
EGFR	Epidermal growth factor receptor
FRAP	Ferric ion reducing power
GLUT4	Glucose transporter-4
MTT	3-(4,5-Dimethylthiazol-2-yl)-2,5-diphenyltetrazolium bromide
mTOR	Mechanistic target of rapamycin kinase
2-NBDG	2-[N-(7-Nitrobenz-2-oxa-1,3-diazol-4-yl) amino]-2-deoxyglucose; PI3K: phosphatidylinositol-3-hydroxykinase
PIK3CA	Phosphatidylinositol-4,5-bisphosphate 3-kinase catalytic subunit alpha
PIK3CB	Phosphatidylinositol-4,5-bisphosphate 3-kinase catalytic subunit beta
PIK3R1	Phosphoinositide-3-kinase regulatory subunit 1
PPI network	Protein-protein interaction network
PTK2	Protein tyrosine kinase 2
RIPA lysis buffer	Radio immunoprecipitation assay lysis buffer
SRC	SRC proto-oncogene, non-receptor tyrosine kinase
STAT3	Signal transducer and activator of transcription 3
