# Supplementary material for: Potential Involvement of PI3K/AKT Signaling Pathway in the Protective Effects of Rhinacanthus nasutus Against Diabetic Nephropathy-Induced Oxidative Stress
Source: Antioxidants (Basel). 2026 Feb 14;15(2):252. doi: 10.3390/antiox15020252 (PMC12938351; doi:10.3390/antiox15020252)
Supplement: Supplementary file 1 [file antioxidants-15-00252-s001.zip › Supplementary material-TableS1.pdf]

Table S1. Tentatively identification of compounds in AE by Q Exactive HF LC-MS.

No	Rt (min)	Identification	Positive ion or Negative ion (m/z)			Element composition	Molecular weight (Da)	MS/MS(m/z)	Compound classification
			M±H	Indicated	ppm				
1	1.111	3',4'-Dihydroxy-7-methoxyflavan	M+H	272.10428	-2.12	$C_{16}H_{16}O_4$	273.09342	$273[M+H]^+$,$240[M+H-CH_3-H_2O]^+$	Flavonoids
2	1.798	2,4-Dihydroxycinnamic acid	M-H	180.04176	-2.75	$C_9H_8O_4$	179.03444	$179[M-H]^-$, $161[M-H-H_2O]^-$, $135[M-H-COO]^-$, $130[M-H-OH-2O]^-$, $117[M-H-COO-H_2O]^-$	Cinnamic acid derivatives
3	3.071	Vanillic acid	M+H	168.04187	-2.30	$C_8H_8O_4$	169.04904	$169[M+H]^+$, $136[M+H-(-OOH)]^+$, $123[M+H-HCOOH]^+$, $118[M+H-CH_7O_2]^+$, $111[M+H-2CHO]^+$	Cinnamic acid derivatives
4	4.763	2,6-Dimethoxyquinone	M+H	193.05046	-1.2	$C_8H_8O_4$	169.04915	$169[M+H]^+$, $118[M+H-CH_7O_2]^+$	Phenolic acid

5	5.298	1-O-(4-coumaroyl)-beta-D-glucose	M-H	326.09922	-2.92	$C_{15}H_{18}O_8$	325.09192	325[M-H]$^-$, 239[M-H-2CHO-CO]$^-$, 231[M-H-C_6H_6O]$^-$, 197[M-H-2CHO-CO-C_2H_2O]$^-$, 179[M-H-2CHO-CO-C_2H_2O-H_2O]$^-$, 167[M-H-2CHO-CO-C_2H_2O-CH_3OH]$^-$	Glycoside
6	5.539	3-[3-(beta-D-Glucopyranosyloxy)-2-hydroxyphenyl]propanoic acid	M-H	344.10966	-3.11	$C_{15}H_{20}O_9$	343.10242	343[M-H]$^-$, 325[M-H-H_2O]$^-$, 239[M-H-H_2O-$C_4H_6O_2$]$^-$, 197[M-H-H_2O-$C_6H_8O_3$]$^-$, 162[M-H-H_2O-$C_6H_{11}O_4$]$^-$	Glycoside
7	6.531	3-(3',5'-Dihydroxyphenyl)propanoic acid	M+H	182.05744	-2.60	$C_9H_{10}O_4$	183.06471	183[M+H]$^+$, 165[M+H-H_2O]$^+$, 149[M+H-H_2O_2]$^+$, 147[M+H-2H_2O]$^+$, 123[M+H-H_2O_2-C_2H_2]$^+$, 118[M+H-2H_2O-CHO]$^+$	Phenolic acid
8	6.636	trans O-Coumaric acid	M+H	164.04690	-2.70	$C_9H_8O_3$	165.05421	165[M+H]$^+$, 147[M+H-H_2O]$^+$,	Cinnamic acid derivatives
9	6.890	4-Methylumbelliferone hydrate	M+H	176.04689	-2.57	$C_{10}H_8O_3$	177.05417	177[M+H]$^+$,163[M+H-CH_2]$^+$,149[M+H-CO]$^+$, 118[M+H-CO-CH_3-O]$^+$	Coumarin derivatives

10	6.908	Syringic acid	M-H	198.0522	-3.15	$C_9H_{10}O_5$	197.04494	197[M-H]$^-$, 179[M-H-H$_2$O]$^-$, 148[M-H-H$_2$O-CH$_3$O]$^-$, 135[M-H-H$_2$O-COO]$^-$	Phenolic acid
11	7.321	Homogentisic acid	M+H	168.04226	-2.30	$C_8H_8O_4$	169.04901	169[M+H]$^+$, 118[M+H-3OH]$^+$	Phenolic acid
12	8.094	p-Hydroxymandelic acid	M+H	168.04178	-2.83	$C_8H_8O_4$	169.04906	169[M+H]$^+$, 118[M+H-3OH]$^+$	Phenolic acid
13	10.022	Esculetin	M+H	178.02609	-2.89	$C_9H_6O_4$	179.03336	179[M+H]$^+$,149[M+H-CH$_2$O]$^+$,118[M+H-COO-OH]$^+$	Coumarin derivatives
14	10.610	Trans Ferulic acid	M+H	194.05745	-2.37	$C_{10}H_{10}O_4$	195.06462	195[M+H]$^+$, 177[M+H-H$_2$O]$^+$, 149[M+H-H$_2$O-CO]$^+$, 118[M+H-H$_2$O-CO-OCH$_3$]$^+$	Phenolic acid
15	10.650	Dihydroferulic acid	M+H	196.07356	-2.85	$C_{10}H_{10}O_4$	197.08028	197[M+H]$^+$, 149[M+H-CH$_4$O]$^+$, 118[M+H-C$_2$H$_7$O$_3$]$^+$	Phenolic acid
16	10.943	3,4',5,6,7-Pentamethoxyflavone	M+H	372.11775	-3.71	$C_{20}H_{20}O_7$	373.12680	373[M+H]$^+$, 344[M+H-CHO]$^+$, 295[M+H-C$_2$H$_6$O$_3$]$^+$, 279[M+H-C$_2$H$_6$O$_4$]$^+$, 269[M+H-C$_2$H$_6$O$_3$-2CH]$^+$, 209[M+H-CHO-C$_8$H$_7$O$_2$]$^+$, 193[M+H-C$_2$H$_6$O$_3$-2CH-C$_6$H$_4$]$^+$, 149[M+H-	Flavonoids

								$C_2H_6O_3$-2CH-C_6H_4-$C_2H_4O]^+$, 118[M+H-$C_2H_6O_4$-$C_{11}H_{13}O]^+$,	
17	11.002	4-Acetyl-3-hydroxy-5-methylphenyl β-D-glucopyranoside	M-H	328.11487	-2.88	$C_{15}H_{20}O_8$	327.10760	327[M-H]$^-$,228[M-H-$C_5H_7O_2]^-$,228[M-H-$C_5H_7O_2$-CH_3-$H_2O]^-$,182[M-H-$C_5H_7O_2$-C_2H_4-$H_2O]^-$,112[M-H-$C_5H_7O_2$-C_2H_4--C_4H_4-2$H_2O]^-$	Glycoside
18	11.328	Benzoic acid, 2-(b-D-glucopyranosyloxy)-, ethyl ester	M+H	328.11452	-3.94	$C_{15}H_{20}O_8$	329.12177	329[M+H]$^+$,279[M+H-CH_3-H_2O-OH]$^+$,241[M+H-$C_4H_8O_2]^+$, 227[M+H-$C_5H_{10}O_2]^+$,167[M+H-$C_5H_{10}O_2$-$C_2H_4O_2]^+$, 149[M+H-$C_5H_{10}O_2$-$C_2H_4O_2$-$H_2O]^+$	Glycoside
19	11.647	m-Coumaric acid	M+H	164.04689	-2.76	$C_9H_8O_3$	165.05417	165[M+H]$^+$, 147[M+H-$H_2O]^+$	Cinnamic acid derivatives
20	12.079	Ferulic Acid	M+H	194.05745	-2.36	$C_{10}H_{10}O_4$	195.06470	195[M+H]$^+$, 177[M+H-$H_2O]^+$, 149[M+H-H_2O-CO]$^+$, 118[M+H-H_2O-CO-$OCH_3]^+$	Phenolic acid
21	12.315	Methyl vanillate	M+H	182.05791	-2.60	$C_9H_{10}O_4$	183.06470	183[M+H]$^+$, 149[M+H-2OH]$^+$, 118[M+H-2OH-$OCH_3]^+$,	Cinnamic acid derivatives

22	12.873	3-Methoxy-phenylacetic acid	M-H	166.06247	-3.17	$C_9H_{10}O_3$	165.05516	165[M-H]$^-$, 121[M-H-COO]$^-$	Phenolic acid
23	12.887	7-Hydroxycoumarine	M+H	162.03119	-3.11	$C_9H_6O_3$	163.03847	163[M+H]$^+$,118[M+H-CO-OH]$^+$	Coumarin derivatives
24	13.209	Sinensetin	M+H	372.11987	-3.71	$C_{20}H_{20}O_7$	373.1201	373[M+H]$^+$, 353[M+H-CH_2]$^+$, 279[M+H-CH_2-$C_2H_8O_3$]$^+$, 223[M+H-$C_9H_{10}O_2$]$^+$, 209[M+H-$C_9H_8O_3$]$^+$, 163[M+H-$C_9H_{10}O_2$-2CO]$^+$, 149[M+H-CH_2-$C_2H_8O_3$-C_9H_6O]$^+$	Flavonoids
25	13.526	Isosinensetin	M+H	372.11952	-3.71	$C_{20}H_{20}O_7$	373.12686	373[M+H]$^+$, 355[M+H-H_2O]$^+$, 279[M+H-H_2O-$C_3H_8O_2$]$^+$, 270[M+H-$C_4H_7O_3$]$^+$, 149[M+H-$C_4H_7O_3$-C_8H_9O]$^+$, 118[M+H-H_2O-$C_3H_8O_2$-$C_{10}H_9O_2$]$^+$	Flavonoids
26	14.529	Coumarin	M+H	146.03635	-2.95	$C_9H_6O_2$	147.04362	147[M+H]$^+$,118[M+H-CHO]$^+$	Coumarins
27	17.344	Cinnamic acid	M-H	148.05200	-2.90	$C_9H_8O_2$	147.04471	147[M-H]$^-$, 134[M-H-CH_2]$^-$, 116[M-H-CH_2-H_2O]$^-$	Cinnamic acids
28	17.428	Salvigenin	M-H	328.09361	-3.28	$C_{18}H_{16}O_6$	327.08633	327[M-H]$^-$, 243[M-H-C_5H_8O]$^-$, 195[M-H-$C_5H_8O_4$]$^-$, 182[M-H-$C_5H_8O_4$-CH]$^-$, 153[M-H-$C_5H_8O_4$-CH-CHO]$^-$, 147[M-	Flavonoids

								H-$C_9H_8O_4$]$^-$, 116[M-H-$C_5H_8O_4$-CH-CHO-C_3H_2]-	
29	19.088	Rhinacanthin A	M+H	258.08827	-3.62	$C_{15}H_{14}O_4$	259.09555	259[M+H]$^+$, 241[M+H-H_2O]$^+$, 209[M+H-H_2O-CH_3OH]$^+$, 192[M+H-H2O-CH_5O_2]$^+$	Naphthoquinone
30	21.897	Ethyl 3-(3,4-dihydroxyphenyl)propionate	M-H	210.08858	-3.01	$C_{11}H_{14}O_4$	209.08128	209[M-H]$^-$, 182[M-H-C_2H_5]$^-$, 112[M-H-C_2H_5-$C_3H_2O_2$]$^-$	Phenolic acid
31	22.116	3-Oxoindane-1-carboxylic acid	M+H	176.04689	-2.57	$C_{10}H_8O_3$	177.05411	177[M+H]$^+$,149[M+H-CO]$^+$,118[M+H-CO-H_2O-CH]$^+$	Phenolic acid
32	25.296	5-Hydroxy-1-(4-hydroxy-3-methoxy-cyclohexyl)decan-3-one	M+H	294.18208	-3.48	$C_{17}H_{26}O_4$	295.18936	295[M+H]$^+$,244[M+H-CH3-2H_2O]$^+$, 227[M+H-CH_3-2H_2O-OH]$^+$,141[M+H-$C_9H_{14}O_2$]$^+$	Organic ketone
33	26.199	Rhinacanthone	M+H	242.09349	-3.31	$C_{15}H_{14}O_3$	243.10078	243[M+H]$^+$, 209[M+H-2OH]$^+$, 192[M+H-3OH]$^+$, 163[M+H-3OH-C_2H_5]$^+$, 149[M+H-3OH-C_2H_5-CH_2]$^+$,	Naphthoquinone

34	26.697	2-(8-Hydroxy-4a,8-dimethyl-decahydro-2-naphthalenyl)acrylic acid	M-H	234.16198	-3.15	$C_{15}H_{24}O_3$	233.15393	233[M-H]$^-$, 215[M-H-H_2O]$^-$, 197[M-H-2H_2O]$^-$, 182[M-H-2H_2O-CH_3]$^-$, 159[M-H-2H_2O-CH_3-CHO]$^-$, 130[M-H-$C_4H_7O_3$]$^-$, 115[M-H-$C_4H_7O_3$-CH_3]$^-$	Sesquiterpenoids
35	26.707	Thujopsenic acid	M+H	234.16123	-3.22	$C_{15}H_{22}O_2$	235.16853	235[M+H]$^+$,209[M+H-2CH]$^+$,192[M+H-2CH-OH]$^+$, 163[M+H-2CH-OH-CHO]$^+$, 149[M+H-2CH-OH-CHO-CH_2]$^+$	Sesquiterpenoids
36	28.272	2,6-Di-tert-butyl-1,4-benzoquinone	M+H	220.14562	-3.24	$C_{14}H_{20}O_2$	221.15306	221[M+H]$^+$,199[M+H-H_2O]$^+$, 181[M+H-2H_2O]$^+$,149[M+H-C-4CH_3]$^+$	Benzoquinones
37	31.284	Caffeic acid	M+H	180.04170	-3.08	$C_9H_8O_4$	181.04889	181[M+H]$^+$, 163[M+H-H_2O]$^+$, 149[M+H-2O]$^+$, 118[M+H-CH_3O_3]$^+$	Phenolic acid
38	31.349	(2-Methyl-heptyl)-malonic acid diethyl ester	M+H	272.19774	-3.74	$C_{15}H_{28}O_4$	273.20508	273[M+H]$^+$,241[M+H-2O]$^+$, 228[M+H-2O]$^+$, 209[M+H-CHO_2]$^+$,118[M+H-$C_8H_{15}O_2$]$^+$	Organic esters
