# Supplementary material for: Potential Involvement of PI3K/AKT Signaling Pathway in the Protective Effects of Rhinacanthus nasutus Against Diabetic Nephropathy-Induced Oxidative Stress
Source: Antioxidants (Basel). 2026 Feb 14;15(2):252. doi: 10.3390/antiox15020252 (PMC12938351; doi:10.3390/antiox15020252)
Supplement: Supplementary file 1 [file antioxidants-15-00252-s001.zip › Supplementary material-TableS2.pdf]

Table S2. The key active ingredients of AEB associated with diabetic kidney disease.

Card	Name	Compound Classification	BetweennessCentrality	ClosenessCentrality	Degree
AEB16	Isosinensetin	Flavonoids	0.027183	0.416667	91
AEB22	Sinensetin	Flavonoids	0.017274	0.401891	76
AEB12	5-hydroxy-1-(4-hydroxy-3-methoxy-cyclo-hexyl)decan-3-one	Phenolic acid	0.023968	0.401891	76
AEB21	Salvigenin	Flavonoids	0.015148	0.399061	73
AEB3	[6]-Gingerdiol	Organic acid	0.021223	0.39627	70
AEB9	3,4',5,6,7-Pentamethoxyflavone	Flavonoids	0.012144	0.39261	66

AEB6	2β,9α-diacetoxy-trans-decalin	Sesquiterpenoids	0.012646	0.382883	55
AEB20	Rhinacanthone	Naphthoquinone	0.01245	0.382022	54
AEB14	Ethyl 3-(3,4-dihydroxyphenyl)propionate	Phenolic acid	0.010757	0.37694	48
AEB2	(2-methyl-heptyl)-malonic acid diethyl ester	Organic esters	0.008884	0.374449	45
AEB7	3-(2,2,5,6-tetramethyl-5-(((2-oxo-2H-chromen-7-yl)oxy)methyl)-1-oxaspiro[2.5]octan-4-yl)propanoic acid	Flavonoids	0.008519	0.371179	41
AEB1	(1aS,4aS,8aR)-4a,8,8-trimethyl-1,1a,4,5,6,7-hexahydrocyclopropa[j]naphthalene-2-carboxylic acid	Sesquiterpenoids	0.007797	0.37037	40
AEB13	Embelin	Phenolic acid	0.004552	0.363248	31
AEB5	2,6-Di-tert-butyl-1,4-benzoquinone	Benzoquinones	0.003571	0.360169	27

AEB4	2,4-dihydroxycinnamic acid	Cinnamic acid derivatives	0.000476	0.349794	13
AEB11	3-Methoxyphenylacetic acid	Phenolic acid	0.00077	0.349076	12
AEB17	O-Coumaric acid	Cinnamic acid derivatives	0.000345	0.348361	11
AEB10	3-Coumaric acid	Cinnamic acid derivatives	0.000338	0.348361	11
AEB18	Phenylpyruvic Acid	Phenolic acid	0.000447	0.346939	9
AEB15	Homogentisic acid	Phenolic acid	0.000243	0.346232	8
AEB19	3,4',5,6,7-Pentamethoxyflavone	Flavonoids	9.87E-05	0.34413	5
AEB8	3, 5-dihydroxybenzaldehyde	Naphthoquinone	0.000143	0.34413	5

2
