# Supplementary material for: Potential Involvement of PI3K/AKT Signaling Pathway in the Protective Effects of Rhinacanthus nasutus Against Diabetic Nephropathy-Induced Oxidative Stress
Source: Antioxidants (Basel). 2026 Feb 14;15(2):252. doi: 10.3390/antiox15020252 (PMC12938351; doi:10.3390/antiox15020252)
Supplement: Supplementary file 1 [file antioxidants-15-00252-s001.zip › Supplementary material-TableS3.pdf]

antioxidants

MDPI

Table S3. The key targets of AEB associated with diabetic kidney disease (Top 10). 1

Gene	Protein	Betweenness Centrality	Closeness Centrality	Degree
SRC	SRC proto-oncogene, non-receptor tyrosine kinase	0.145376888	0.37191358	47
PIK3R1	Phosphoinositide-3-kinase regulatory subunit 1	0.036095551	0.344778255	37
PIK3CA	Phosphatidylinositol-4,5-bisphosphate 3-kinase catalytic subunit alpha	0.021257052	0.338483146	35
PIK3CB	Phosphatidylinositol-4,5-Bisphosphate 3-Kinase Catalytic Subunit Beta	0.014549278	0.322623829	34
STAT3	Signal transducer and activator of transcription 3	0.099692771	0.371340524	34
HSP90AA1	Heat shock protein 90 alpha family class A member 1	0.153323594	0.374805599	31
AKT1	AKT serine/threonine kinase 1	0.059069265	0.362951807	29
PTK2	Protein Tyrosine Kinase 2	0.015064787	0.322623829	27

2				
EGFR	Epidermal growth factor receptor	0.052879142	0.348769899	26
ITGB1	Integrin Subunit Beta 1	0.073234696	0.314211213	26
